# Human bone marrow derived stem cell differentiation on 3D printed bioactive glass scaffolds

**DOI:** 10.1007/s10856-025-06918-y

**Published:** 2025-08-27

**Authors:** Siwei Li, Ali A. Mohammed, Amy Nommeots-Nomm, Xiaomeng Shi, Fadi Barrak, Agathe Heyraud, Julian R. Jones

**Affiliations:** 1 Department of Materials, Imperial College London, London, UK; 2 VSS Academy Training & Education Ltd, London, UK; 3 Dyson School of Design Engineering, Imperial College London, London, UK; 4 School of Design, Royal College of Art, London, UK

## Abstract

Bioactive glass particles have previously been found to stimulate new bone growth in vivo and have a long clinical track record. The effect of bioactive glasses on human bone marrow derived stromal cells (hBMSCs) has not been clearly ascertained previously. Recently, 3D printed scaffolds of the ICIE16 glass composition (49.46 mol% SiO_2_, 36.6 mol% CaO, 6.6 mol% Na_2_O, 6.6 mol% K_2_O, 1.07 mol% P_2_O_5_) were found to produce high quality bone ingrowth in vivo in a rabbit model. This composition was chosen because it can be sintered into scaffolds without crystallisation. Here, we cultured hBMSCs on the 3D printed ICIE16 scaffolds to determine whether the scaffolds can support cell growth and osteogenic differentiation in vitro, with and without the presence of osteogenic supplements. This was compared to a control of culture media containing dissolution products of the bioactive glass scaffold. Our hypothesis was that the cells cultured on the scaffolds would undergo more osteogenic differentiation than cells cultured in media containing only the dissolution ions of the scaffolds, even without osteogenic supplements. hBMSCs cultured on ICIE16 scaffolds significantly increased expression of osteogenic differentiation and matrix formation markers, including Runx 2, Col1a1, Osteopontin, Osteocalcin and Alkaline Phosphatase, in comparison to monolayer cultures in basal conditions with bioactive glass dissolution products, at all time points up to 6 weeks. Six weeks was chosen as it is the time scale for bone fracture healing. The presence of osteogenic supplements appeared to have synergetic effects with 3D scaffolds, especially during early stages of osteogenic differentiation (week 2 and 4). By week 6, there was no significant difference in the expression of osteogenic markers by hBMSCs cultured on ICE16 scaffolds with and without osteogenic supplements. These findings support our hypothesis and highlight that the 3D structure and the dissolution of ICIE16 bioactive glass ionic products both independently influence osteogenic differentiation of hBMSCs.

**Graphical Abstract:**

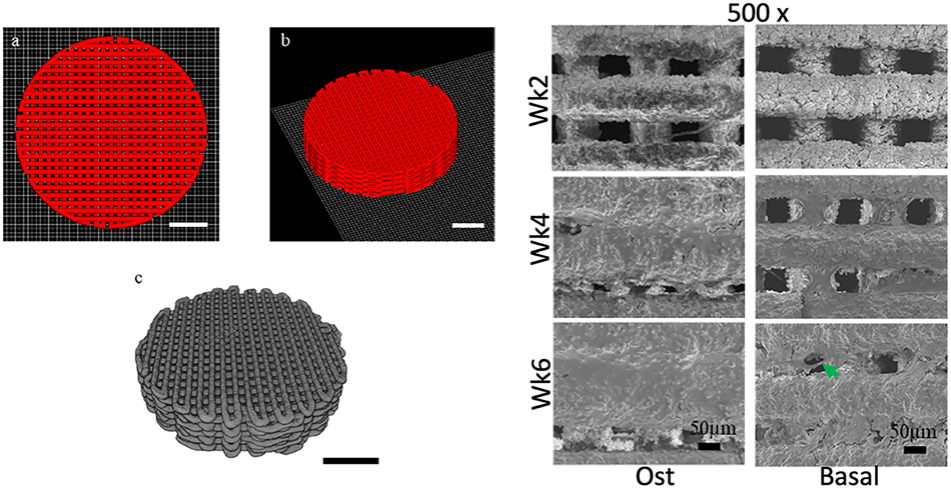

## Introduction

Bioactive glass products have been successful in orthopaedic and dental applications due to their bone-bonding ability [1–3]. The majority of bioactive glass medical devices for orthopaedic indications are based on particulates of the original 45S5 Bioglass^®^ (46.1 mol% SiO_2_, 24.4 mol% Na_2_O, 26.9 mol% CaO, and 2.6 mol% P_2_O_5_) composition [4]. The osteogenic properties of bioactive glass were attributed to the dissolution products of the glass, specifically soluble silica species and calcium ions. Experiments in which primary human osteoblasts (HOBs) were cultured in medium conditioned with the dissolution products of 45S5 powder found enhanced expression of osteoblast mitogenic growth factor and insulin-like growth factor II (IGF-II) [5]. The conditioned medium was also found to enhance the cytosolic calcium concentration inside osteoblasts and adenosine triphosphate (ATP) production [6]. Xynos et al. reported up-regulation of genes related to osteoblast metabolism and bone homoeostasis (via cDNA microarray profiling), e.g. metalloproteinases such as *MMP****2*** and *MMP14* involved in extracellular matrix remodelling [1].

The effect of bioactive glass and its dissolution products on osteogenic differentiation of human bone marrow derived stromal cells (hBMSCs), most commonly referred to as mesenchymal stem cells (hMSCs), remains debatable [7–13]. In part, this may be because hBMSCs, without specific selection based on surface markers, contain a variety of mature, progenitor and stem cells and varies greatly depending on the donor [14, 15]. Studies have demonstrated that hMSCs cultured on 45S5 glass discs or in the presence of dissolution products, did not produce consistent osteogenic responses [16], whereas other studies demonstrated that dissolution products of bioactive glass with different composition and dosage resulted in distinct angiogenic and osteogenic responses from hMSCs [17, 18].

Internalisation of nanoparticles enables intracellular delivery of ions. When bioactive glass nanoparticles containing only SiO_2_-CaO where internalised by hBMCs, the cells did not differentiate, but when the particles also contained strontium (SiO_2_-CaO-SrO), they did [19]. However, SiO_2_-CaO particles did have an osteogenic effect on rat bone marrow derived mesenchymal stem cells [20]. It is possible, however, positive effects of bioactive glass on bone growth in human patients are not solely mediated by accelerated differentiation of stem cells, but rather a combination of 3D environment and other repairing cell populations.

While the some bioactive glass compositions, such as 45S5 and S53P4 (53.8 mol% SiO_2_, 21.8 mol% CaO, 22.7 mol% Na_2_O, 1.7 mol% P_2_O_5_), have found commercial success as particulates, or in putties [4], scaffolds are difficult to produce due to crystallisation of the glasses during scaffold production. This is because scaffold production from glass particles usually involves a sintering process and sintering 45S5 and S53P4 compositions leads to crystallisation, producing a glass-ceramic [21]. New compositions, such as ICIE16 (49.46 mol% SiO_2_, 36.6 mol% CaO, 6.6 mol% Na_2_O, 6.6 mol% K_2_O, 1.07 mol% P_2_O_5_), were developed to increase the temperature difference between the glass transition temperature of the glass and its onset of crystallisation temperature [22], and enable production of porous scaffolds that retained the amorphous glass structure [23]. 3D printing of the ICIE16 composition enabled production of scaffolds with high compressive strength and open pore channels [24] that showed high quality bone regeneration in vivo [25]. In another study, it was reported that surface functionalisation of ICIE16 by nitridation with ammonia gas increased the rate of calcium phosphate or apatite deposition, which in turn resulted in improved MC3T3 cell proliferation and ALP activity [26].

Our hypothesis was that ICIE16 ionic dissolution products would not stimulate osteogenic differentiation in monolayer cultured hBMSCs, but differentiation would be seen if the cells were seeded in direct contact between the cells and glass itself as well as a continual release of ions. Although ICIE16 scaffolds have previously been 3D printed, the aim here was to produce scaffolds with consistent pore channel size and strut size and investigate the osteogenic potential of the scaffolds by using them as templates for in vitro synthesis of bone tissue. While ideal pore architecture is not known, it is known that the pores should be sufficiently open to allow cell migration and vascularised bone ingrowth, e.g. greater than 100 μm.

## Methods and materials

Cell culture consumables and reagents were purchased from Thermo Fisher Scientific (Paisley, UK) and Sigma-Aldrich (Dorset, UK) unless specified otherwise.

### Sample preparation

Glass powders ( < 32 µm diameter) were produced by grinding and sieving melt derived ICIE16 glass frit (49.46 mol% SiO_2_, 36.6 mol% CaO, 6.6 mol% Na_2_O, 6.6 mol% K_2_O, 1.07 mol% P_2_O_5_). To produce the frit, reagents of high purity silica (SiO_2_) (Prince minerals, Stoke-on-Trent), phosphorous pentoxide (P_2_O_5_) and the carbonate equivalent of the modifying oxides were mixed for 8 h (Wheaton mini roller, UK) prior to melting at 1400 °C for 1.5 h in a Pt-5%Au crucible [23]. The melt was quenched into deionised water, the glass frit was collected and dried at 100 °C prior to being ball milled and sieved to yield particles with diameter less than 32 μm, which is the finest size that can be produced by standard ball milling.

**Fig. 1:**
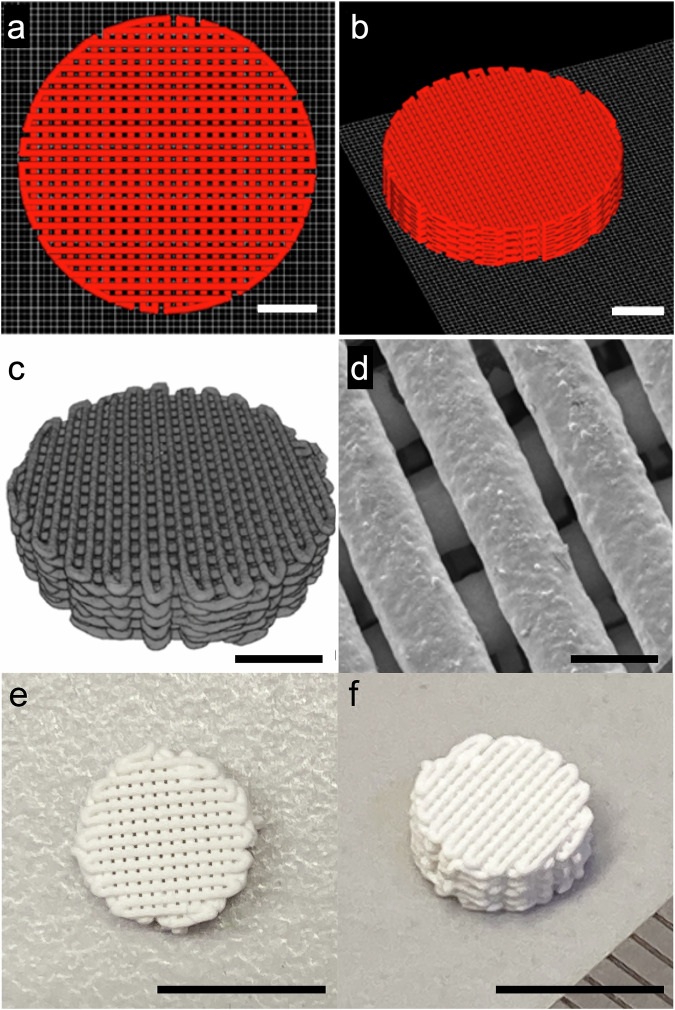
**a**, **b** 3D images of scaffold designs for Direct Ink Writing, created in RoboCAD 3.0 (3Dinks, Tulsa, USA) software: **a** top view; **b** side view; **c** 3D rendered image of X-ray microtomography scans of a 3D printed bioactive glass scaffold after sintering. Scale bars (**a**–**c**) = 2 mm; **d** SEM image of an ICIE16 scaffold after sintering; **e**, **f** photograph of scaffold, scale bar = 200 μm (**a**–**d**), 1 cm (**e**, **f**)

The ICIE16 bioactive glass scaffolds were 3D printed by Direct Ink Writing at glass to ink ratio of 45% (v/v) in a solution of 25% (w/v) Pluronic F-127, following previous studies [24]. Scaffolds were printed using a Robocaster system (3DInks, Tulsa, USA) with a built-in RoboCAD 3.0 (3Dinks, Tulsa, USA) software. Smooth-flow tapered tips with nozzle diameter 250 μm were used. The scaffolds were designed to have circular shape (9 mm diameter × 2 mm height), with strut size 250 μm and spacing 250 μm (prior to shrinkage and sintering). The scaffolds were sintered at 700 °C for 3 h. All sintering processes were conducted at a heating rate of 3 °C min^–1^ [23]. Figure 1a, b shows the initial design of the scaffolds and the structure of scaffolds after 3 h sintering Fig. 1c.

### Surface analysis preparation

The scaffolds were dehydrated through a series of increasing concentrations of ethanol (70%, 80%, 90%, and 100%) and coated with gold for the scanning electron microscopy (SEM) with energy dispersive X-ray spectroscopy (EDS). Images and EDS spectrum were acquired using a LEO 1525 Field Emission SEM (Zeiss, Germany). The cultured scaffolds for fracture surface analysis were embedded in LR white resin (TAAB Laboratories Equipment Ltd, UK) after dehydration for sectioning. The scaffolds were cut using a 0.3 mm thick IsoMetTM Diamond Wafering Blades (Buehler, USA) through the centre of the scaffolds. The sectioned surfaces were then ground successively with K800, K2000, and K4000 grinding paper, followed by a chromium coating. For X-ray diffraction (XRD) analysis, the dehydrated samples were ground into powder. The glass powder samples were place on an amorphous silicon disk to provide a zero background. Diffraction patterns were collected with a Philips X’PERT PRO MPD between 5° and 80° 2θ angle, with step size 0.0334° and step time 100 s. The device was set at 40kv and 40 mA.

### Skeletal modulus

Compression tests were performed as previously described [23]. The scaffold density was calculated by measuring the volume and the weight of the scaffolds (scaffold density = ⍴_sc_ = m_sc_ / V_sc_). The percentage porosity was calculated as % porosity = (1 - ⍴_sc_ / ⍴_sk_) * 100%, where a skeletal density of 2.7 g.cm^–3^ was used [27, 28]. Skeletal modulus of the scaffolds (individual struts) was calculated based on bulk scaffold modulus and scaffold porosity. The modulus of each strut was then calculated using the Ashby-Gibson equation (cellular solid theory).

### Cell culture

Bone marrow-derived stromal cells, hBMSCs (ATCC^®^ PCS-500-012™, Teddington, UK), were expanded in T-125 cell culture flasks in basal α-MEM supplement with 10% (v/v) foetal bovine serum (FBS), 100 U mL^–1^ penicillin and 100 μg mL^–1^ streptomycin in humidified atmosphere at 37°C and 5% CO_2_. Cells with passage 2–4 were used.

### Sterilisation and conditioned media

Scaffolds were sterilised with 70% ethanol and washed in phosphate-buffered saline (PBS) before use in cell studies. 75 mg of ICIE16 powders were soaked in 50 mL minimum essential medium alpha modification (α-MEM) for 72 h at 37 °C on a rotator and dissolution products were filter sterilised.

### Cell seeding and culture on 3D scaffolds

For osteogenic differentiation studies, hBMSCs were harvested and suspended in basal media at a concentration of 1 × 10^6^ cells in 1 mL. For controls, 1 mL of cell suspension was added to 6-well plates and cultured in ICIE16 dissolution products with basal supplements. For culture on scaffolds, 1 mL of cell suspension was added to each sterile 50 mL Falcon tube containing one scaffold. The tubes were placed in an incubator for 2 h with gentle agitation every 30 min to allow diffused cell adhesion. The solution was then replaced with fresh basal media or osteogenic media (basal α-MEM supplemented with 100 μM ascorbate-2-phosphate, 10 nM dexamethasone and 10 mM of β-glycerophosphate). Cultures were maintained in humidified atmosphere at 37 °C, 5% CO_2_ and 21% O_2_ for up to 6 weeks with media changes every 3–4 days.

### Analysis of gene expression

At each time point, cells were lysed for extraction of RNA using a Qiagen RNeasy kit (Qiagen, Manchester, UK) following manufacturer’s instructions. The RNA samples were reverse transcribed using a SuperScript^®^ VILO^TM^ cDNA synthesis kit. SYBR green based qPCR assays were carried out for the analyses of osteogenic gene expression, including Runx2 (F: 5’- gtagatggacctcgggaacc -3’; R: 5’- gaggcggtcagagaacaaac -3’), Col1a1 (F: 5’- gagtgctgtcccgtctgc -3’; R: 5’ - tttcttggtcggtgggtg -3’), Osteopontin (F: 5’- gtttcgcagacctgacatcc -3’; R: 5’- cattcaactcctcgctttcc -3’) and Osteocalcin (F: 5’- ggcagcgaggtagtgaagag -3’; R: 5’- ctcacacacctccctcctg -3’) [29]. The expression of genes of interest was normalised to the endogenous control, β-Actin (F: 5’- ggcatcctcaccctgaagta -3’; R: 5’ - aggtgtggtgccagattttc -3’). The relative transcript levels of genes of interest were analysed using the comparative C_T_ method (ΔΔC_T_ method). For each gene of interest, week-2 hBMSCs cultured as monolayer in ICIE16 dissolution products was assigned to a value of 1 and expression levels in the remaining groups were calculated as relative fold increases. Statistical analysis was performed at the level of ΔC_T_.

### Alkaline phosphatase (ALP) assay

ALP activities in cultures were assessed at each time point using p-nitrophenyl phosphate (pNPP). In brief, cell cultures were lysed in distilled water containing 0.9% Triton X-100. Cell lysate (100 μL) was reacted with 1 mg mL^-1^ (2 mM) p-nitrophenyl phosphate dissolved in 0.1 M glycine buffer with 0.1 mM ZnCl_2_ and 0.1 mM MgCl_2_ (pH adjusted to 10.4) (100 μL for 5 min). The reaction was stopped with 1 M NaOH and the light absorption was determined using a microplate reader at 405 nm wavelength.

### Statistical analyses

Results were presented as mean ± S.D. Statistical analysis was performed using Kruskal-Wallis test with Dunn’s post test (3 or more groups) in Prism 7. Results were deemed significant if the probability of occurrence by random chance alone was less than 5% (i.e. p < 0.05).

### Immunohistochemistry

Cultures were fixed with 4% paraformaldehyde (PFA) and used for immunohistochemical analysis of osteogenic differentiation. After permeabilisation with buffered 0.5% (v/v) Triton X-100 in PBS (300 mM sucrose, 50 mM NaCl, 3 mM MgCl_2_, 20 mM Hepes and pH 7.2) and blocking with 10 mg mL^-1^ BSA in PBS, samples were incubated with relevant diluted primary antibody at 4 °C overnight. This was followed by hour-long incubation with Alexa Fluor^®^ 488-conjugated secondary antibody. The anti-Collagen Type I antibody (rabbit polyclonal, IgG, Abcam, Cambridge, UK), anti-Osteopontin antibody (rabbit polyclonal, IgG, Merck Millipore, Watford, UK) and anti-Osteocalcin antibody (rabbit polyclonal, IgG, Merck Millipore, Watford, UK) were used at 1:1000, 1:500 and 1:50 dilutions respectively. Alexa Fluor^®^ 488-conjugated secondary antibody (goat anti-rabbit, IgG, Abcam, Cambridge, UK) was used at a dilution of 1:1000. All samples were counter-stained with DAPI (0.1 μg mL^-1^ in PBS). Negative controls (no staining) were performed with secondary antibody only as well as plain scaffold samples. Stained samples were imaged under confocal microscopy (Leica SP5 MP laser scanning confocal microscope and software, Leica Microsystems, Wetzlar, Germany).

## Results

The scaffolds were printed by direct ink writing glass particles in a polymer carrier (Fig. 1), using CAD designs (Fig. 1a, b). During sintering, polymer was burned out and particles fused, causing shrinkage, so the strut size and spacing reduced from 250 μm to 230 ± 11 μm and from 250 μm to 140 ± 7 μm respectively (Fig. 1c). Scaffold diameter decreased from 9 mm to 7.8 ± 0.2 mm and height reduced from 2 mm to 1.9 ± 0.1 mm (Fig. 1d). Previous studies that monitored the sintering of direct ink written glass scaffolds, using in situ X-ray microtomography, showed that intrastrut porosity was expected, due to incomplete sintering [30]. Here, mean modulus for scaffold struts, based on Ashby-Gibson equation, was 91.93 ± 23.32 MPa, which implies intrastrut porosity was also present in these scaffolds.

**Fig. 2:**
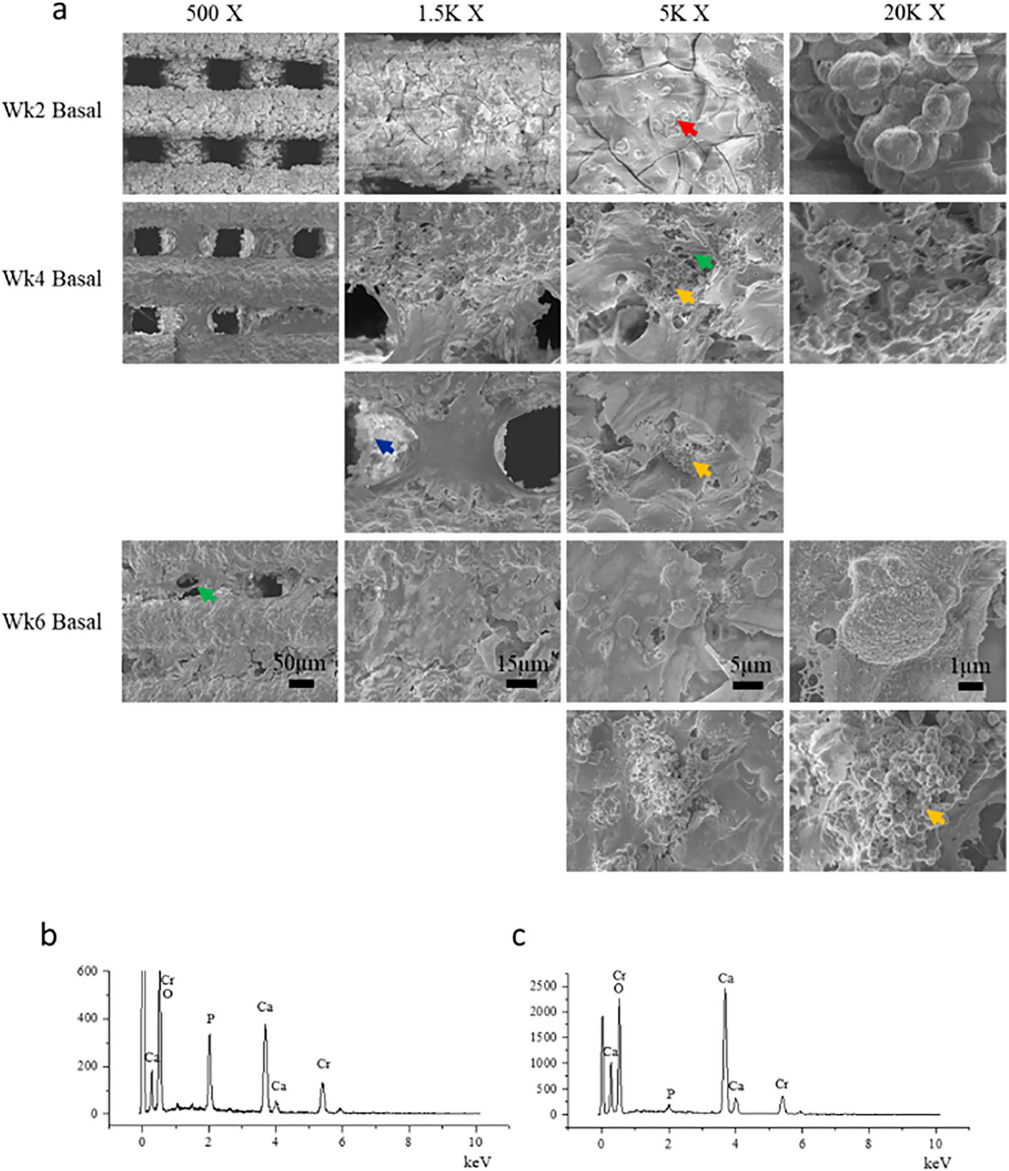
**a** SEM images (500–×20,000 magnification) of surface of 3D printed ICIE16 glass scaffolds cultured with hMSCs under basal conditions for up to 6 weeks: HCA layer (red arrow); bone nodules (yellow arrow); extracellular matrix (green arrow); and calcium carbonate crystals (blue arrow) were visible from week 2; **b** SEM-EDS spectrum of HCA layer; **c** SEM-EDS point spectrum of calcium carbonate. The number above the images represent the magnification factor of images

**Fig. 3:**
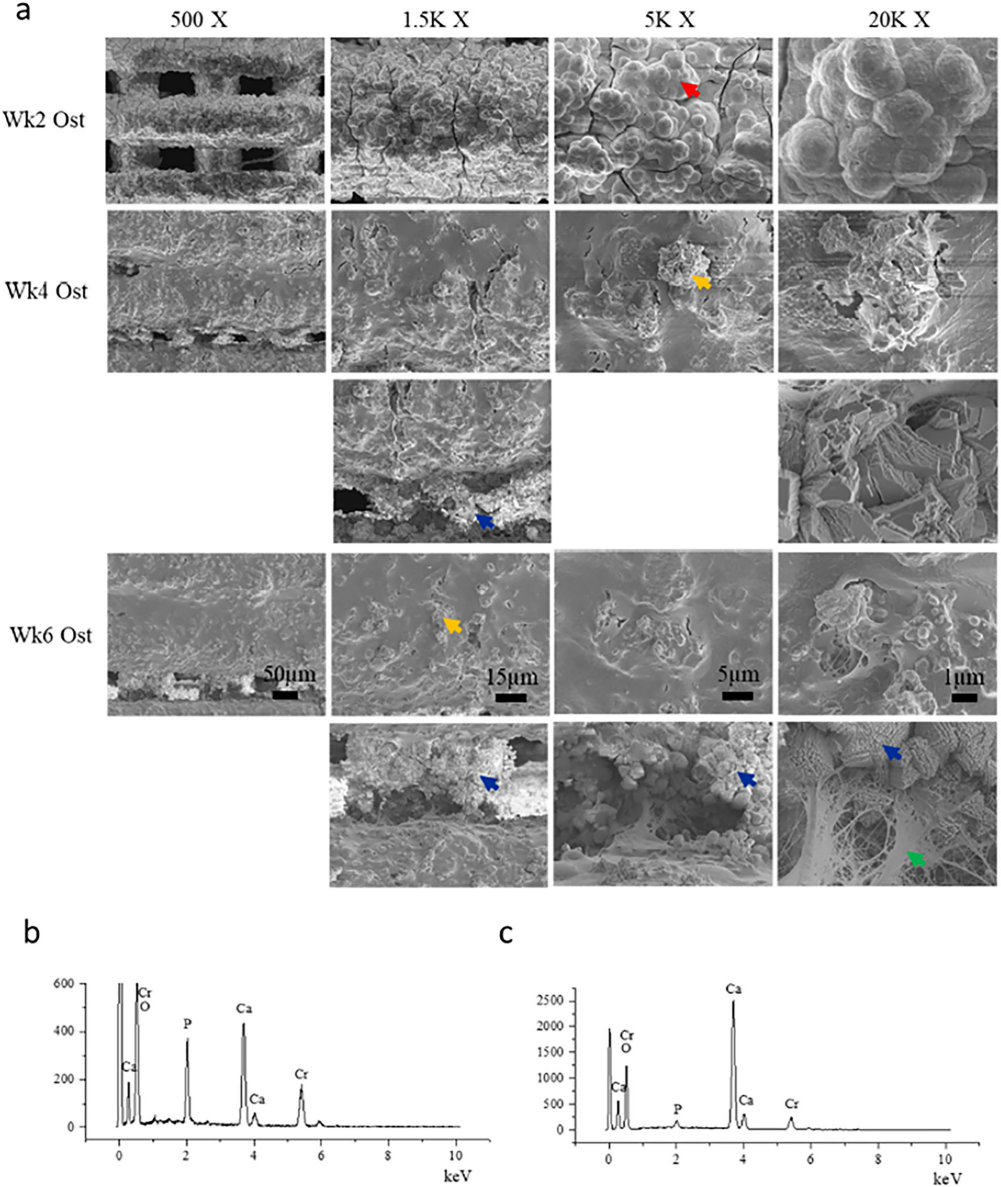
**a** SEM images (500–×20,000 magnification) of surface of ICIE16 glass scaffolds cultured under osteogenic conditions with hMSCs for up to 6 weeks: HCA layer (red arrow); bone nodules (yellow arrow); extracellular matrix (green arrow); and calcium carbonate crystals (blue arrow) were visible from week 2; **b** SEM-EDS spectrum of HCA layer; **c** SEM-EDS spectrum of calcium carbonate. The number above the images represent the magnification factor of images

**Fig. 4:**
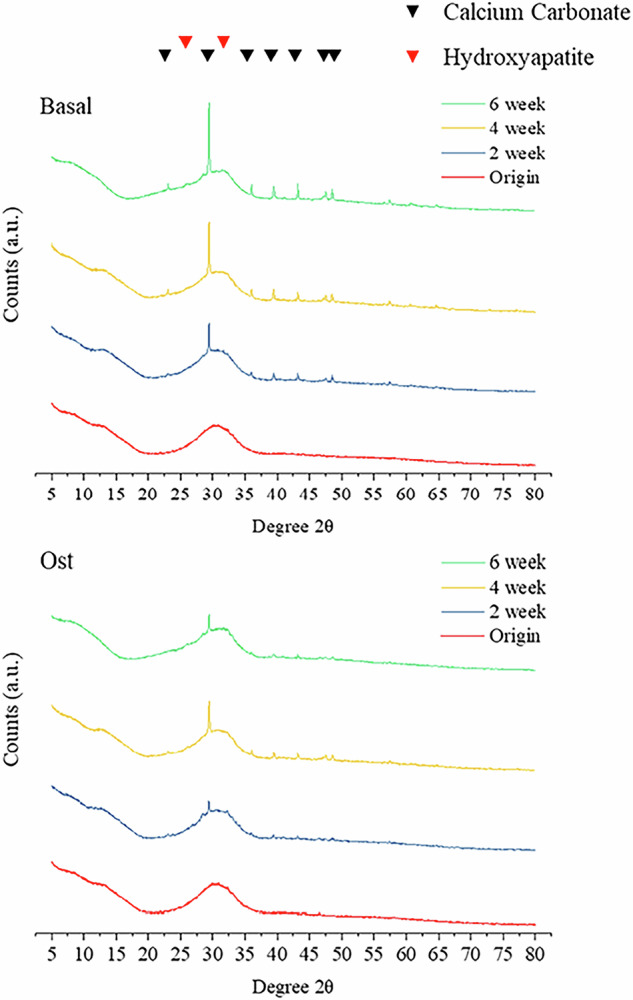
XRD patterns of ICIE16 glass scaffolds after culture with hMSCs in basal and osteogenic cell culture media after 0, 2, 4, and 6 weeks. ICCD reference codes: HA 9-432; calcium carbonate (calcite) 01-072-1937

For in vitro assessment of osteogenic differentiation and matrix formation, hBMSCs were cultured as monolayers in the presence of ICIE16 bioactive glass dissolution products or directly onto printed ICIE16 scaffolds for 6 weeks under basal or osteogenic conditions. Six weeks was chosen because that is the time scale for bone fracture healing. After scaffolds were cultured for 2 weeks in both basal media and osteogenic media, mineral appeared on the scaffold surface which can be seen from the SEM images of the surface of the scaffolds (Figs. 2a, 3a). The mineral forms after ion exchange of K^+^, Na^+^ and Ca^2+^ from the glass with H^+^ from solution, elevating the pH of the culture media [31]. The mineral was confirmed as calcium and phosphorus rich by SEM-EDS (Fig. 2b), by the presence of Ca and P peaks. XRD showed the mineral features to be a mixture of hydroxyapatite (HCA) and calcium carbonate (Fig. 4). Calcium carbonate was identifiable in SEM images from its characteristic faceted morphology, whereas the calcium phosphate rich areas (HCA) were cauliflower-like morphology. After 4 weeks, attached hMSCs cells were observed and they spread on the surface of ICIE16 glass scaffold surface forming cellular multilayers in both basal (Fig. 2a) and osteogenic conditions (Fig. 3a). Their morphology was representative of osteoblasts producing extracellular matrix, indicated by the green arrows. The SEM images showed the presence of discrete 3D cellular bone nodule-like structures (indicated by yellow arrows) throughout the surface of ICIE16 glass scaffolds from week 4 onwards in both groups. These bone nodules were made by a network of cells exhibiting overlapping and superimposed borders and interconnected processes [32]. Besides cauliflower-like HCA layer formation on the surface of the scaffold surface, calcium carbonate crystals (blue arrows) were observed in the SEM images from week 4 onwards, due to calcium ion dissolution products reacting with CO_2_ environment of the incubator.

**Fig. 5:**
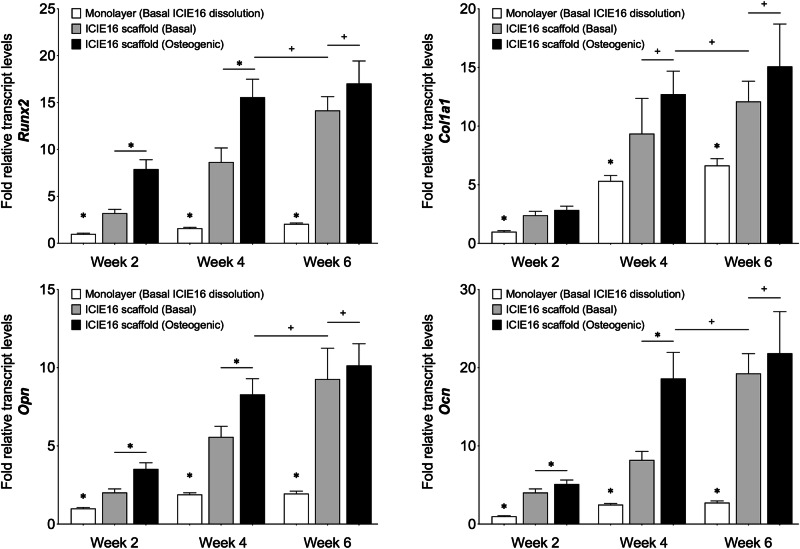
qPCR analysis of gene expression of hBMScs, to investigate the hypothesis that the scaffold plays a role in cell differentiation, not only the dissolution ions, displayed as fold increase of transcription levels relative to hBMSCs cultured as monolayers in ICIE16 dissolution products for 2 weeks, which was assigned to a value of 1. hBMSCs cultured as monolayer in medium containing ICIE16 dissolution products without other osteogenic supplements did not demonstrate capacity for osteogenic differentiation over the 6-week culture period. hBMSCs cultured on the bioactive glass scaffolds without additional osteogenic supplements showed increased transcription of *Runx2*, *Col1al*, *OPN* and *OCN* compared to monolayer culture at each time point. At weeks 2 and 4, cells cultured on scaffolds with additional supplements showed higher transcription than cells cultured on scaffolds alone. By week 6, the expression level of osteogenic markers by hBMSCs cultured on 3D printed ICIE16 scaffolds in basal conditions is similar to those in osteogenic conditions. * indicates p < 0.05 and + indicates p > 0.05

**Fig. 6:**
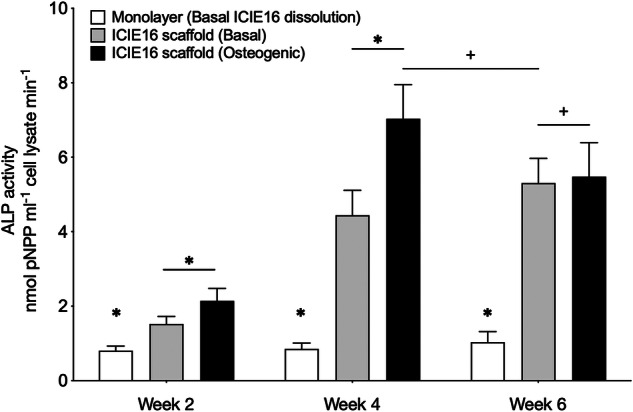
Quantitative ALP assay of hMSCs. The expression of ALP followed a similar trend to qPCR analyses of osteogenic genes. Minimal ALP was detected in monolayer cultured hBMSCs in ICIE16 dissolution products (control), which changed little over 6 weeks. When cells were cultured on scaffolds, the ALP activity increased at all time points. hBMSCs cultured on 3D printed ICIE16 scaffolds in basal conditions expressed ALP at a level comparable to those cultured on scaffolds in osteogenic media. * Indicates p < 0.05 and + indicates p > 0.05

Cells were harvested at week 2, 4 and 6 for gene expression, alkaline phosphatase and immunohistochemical analyses. The hBMSCs cultured as monolayer (without scaffolds) in basal media conditioned with ICIE16 dissolution products did not demonstrate signs of osteogenic differentiation as the expression of key osteogenic markers remained largely unchanged throughout the 6-week culture period (Fig. 5) including Runx2, a marker for osteoblastic differentiation; osteopontin, an indicator of bone-like matrix formation; and osteocalcin, which reflects osteoblastic function and bone turnover. When the hBMSCs were cultured on 3D printed ICIE16 scaffolds, the expression of all osteogenic markers tested were significantly upregulated. The expression level of osteogenic markers from hBMSCs cultured on ICIE16 scaffolds in the presence of osteogenic supplements peaked by week 4. By week 6, hBMSCs cultured on ICIE16 scaffolds in basal conditions showed upregulated expression of all osteogenic markers to a level similar to those cultured on the scaffolds in osteogenic conditions (Fig. 5). Similar observations were made from the expression of ALP, which was determined using a pNNP based colorimetric assay (Fig. 6), with ALP expression increasing when cultured on the scaffolds, peaking at week 4 when osteogenic media was also used, but expression caught up by week 6.

**Fig. 7:**
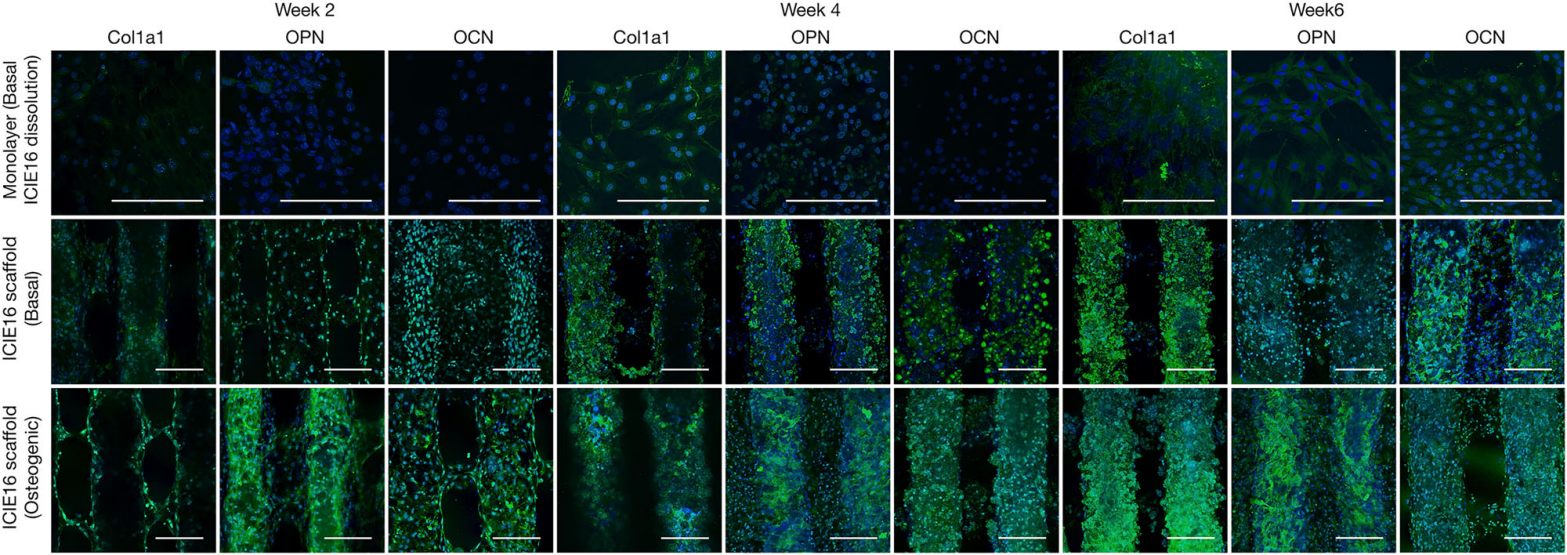
Confocal fluorescence microscopy images of immunohistochemical staining of osteogenic markers, including Collagen Type I (Col1a1), Osteopontin (OPN) and Osteocalcin (OCN) on hBMSCs cultured in monolayers in media containing the bioactive glass dissolution products (control) in basal media; cells cultured on the ICIE16 3D printed scaffolds in basal media; and cells cultured on the scaffolds in osteogenic media. The staining supplements the data from qPCR gene expression analyses and ALP assay. ICIE16 dissolution products and monolayer culture did not support the osteogenic differentiation of hBMSCs. hBMSCs cultured on 3D printed ICIE16 scaffolds were able to synthesise bone-like extracellular matrix without the presence of osteogenic supplements. Green = markers. Blue = cell nuclei. Scale bar = 200 µm

Immunohistochemical analyses of bone matrix markers confirmed the observations from qPCR and ALP assay. Negligible Collagen Type I, Osteopontin and Osteocalcin protein was detected in monolayer cultured hBMSCs in the presence of basal ICIE16 dissolution products (Fig. 7). hBMSCs on 3D printed ICIE16 scaffolds were able to form bone-like matrix under osteogenic condition by week 4, as indicated by positive staining for all the markers from week 2, increasing in intensity over time. By week 6, there was no visual difference between matrix synthesised by hBMSCs cultured on ICIE16 scaffolds in basal conditions and those in osteogenic conditions.

## Discussion

Several alternatives to the original 45S5 Bioglass have been developed with the aim of improved biological and mechanical properties. ICIE16 was chosen here as its network connectivity is similar to 45S5, but it can be sintered without crystallising, thus allowing the possibility of manufacturing 3D printed scaffolds. Previous studies suggested ICIE16 outperformed original 45S5 in promotion of enhanced ALP activity from hMSCS, which is a marker for osteoblastic differentiation [33]. This work shows that the ICIE16 scaffolds can direct hMSc cell differentiation down an osteogenic route, in the absence of osteogenic supplements, and cell attachment to the surface is important, in addition to ion release, and that culturing in osteogenic supplements may offer further synergistic acceleration of bone production in vitro.

Previous in vivo pilot study demonstrated that gel-cast ICIE16 foams promoted sustained bone ingrowth in a femoral head defect rabbit model [23]. In order to further understand the potential of ICIE16 based scaffolds for bone regeneration, in vitro osteogenic differentiation, and subsequent bone-like matrix formation by hBMSCs cultured either as monolayer in the presence of ICIE16 dissolution productions or on 3D printed ICIE16 scaffolds were assessed in the current study. The results from qPCR gene expression analyses, ALP assay and immunohistochemistry (Figs. 5–7) all demonstrated sub-optimal osteogenesis when hBMSCs were cultured as monolayer in the presence of ICIE16 dissolution products alone. The results presented here highlighted the importance of functional 3D structure in tissue engineering and regenerative medicine. hBMSCs cultured on 3D printed ICIE16 scaffolds could undergo osteogenic differentiation and form bone-like matrix without the presence of commonly used osteogenic supplements. This was likely due in part to the porous and interconnected structure in 3D scaffolds. ICIE16 scaffolds used in this study had average spacing of 140 ± 7 μm following sintering. Previous studies have suggested that pores of 100-500 μm could enhance new bone formation, ingrowth and capillary formation [34, 35]. Further studies may be required to investigate the effect of varying manufacturing parameters on pore size, geometry and porosity, which in turn affect not only scaffold mechanical properties and degradation but also cellular behaviour in tissue regeneration applications [36, 37].

There is increasing evidence that microenvironment stiffness and cell-matrix interactions play key roles in lineage specification of naive stem cells [38–42]. There is body of evidence that substrate stiffness alone can determine cell fate and tissue development, where bone marrow derived stem cells tend to differentiate into chondrocytes or adipocytes on low stiffness materials, while high stiffness materials induce osteogenic differentiation [40, 43]. The estimated modulus of individual struts with in the ICIE16 scaffolds was 91.93 ± 23.32 MPa. In contrast, tissue culture plastic has a modulus of 1 × 10^7 ^kPa (Merck / Sigma-Aldrich^TM^). The higher stiffness of ICIE16, coupled with 3D structure, likely acted as a mechanical cue for the osteogenic differentiation of stem cells. This was evidenced in significantly higher expression of osteogenic markers as well as ALP (Figs. 5–7) by cells cultured on ICIE16 scaffolds compared to those on tissue culture plastics. Similarly, previous studies have demonstrated increased osteogenic capacity in primary osteoblasts as well as adipose derived stromal cells, from elderly donors, cultured in 3D environments in comparison to 2D environments, further highlighting the importance of 3D environments in bone tissue engineering [44, 45].

In the current study, the expression level of osteogenic and osseus matrix markers by hBMSCs cultured on ICIE16 scaffolds in basal conditions at week 6 was comparable to those in osteogenic conditions, however, at earlier point (week 4) the expression level under osteogenic conditions was significantly higher (Fig. 5). Similarly, the expression of ALP, a marker for early osteoblast differentiation which elevates during osteogenic differentiation, in hBMSCs under osteogenic conditions also peaked at week 4. These observations suggest a possible synergetic effect of bioactive glass and osteogenic supplements. A previous study also suggested synergy between bioactive glass and mechanical stimulus by fluid perfusion [46]. Other scaffold properties such surface modification [47, 48] and topography [49, 50] have also been demonstrated to be influential in stem cell differentiation, these will need to be investigated further in future studies.

Bioactive glass has been shown to be proangiogenic, albeit in vitro, stimulating the expression of VEGF from fibroblasts and proliferation of microvascular endothelial cells [51]. Ionic dissolution products such as soluble silica, were shown to stimulate both osteogenic gene expression in human osteoblasts and expression of pro-angiogenic markers such as VEGF and VE-cad [32, 52]. The soluble silica species are likely to be SiO_4_^4-^ species, alone or in rings [53]. VEGF is known to influence skeletal development and postnatal bone repair [54], suggesting there may also be paracrine effect between angiogenesis and osteogenesis from bone marrow stromal cells in response to bioactive. In addition to mechanical properties and release of dissolution ions, bioactive glass is also proposed to influence osteogenesis through surface chemistry and topography [55]. Reactions on the surface of silicate bioactive materials induce the release and exchange of critical concentrations of soluble Si species, Ca, P and Na ions, which in turn can lead to favourable intracellular and extracellular responses promoting rapid bone formation [1, 27]. It has been shown that 45S5 promotes human osteoblast proliferation by reducing the growth cycle to pass through G1 and S phase and then enter G2 quickly. In the presence of Si species and Ca ions, osteoblasts that are capable of differentiating into a mature osteocyte phenotype begin to proliferate and regenerate new bone as soon as within 48 h [56]. ICIE16 glass may influence osteogenesis in a similar mechanism, further studies are required.

## Conclusions

The ICIE16 glass scaffolds were successfully fabricated using a direct ink writing technique to generate cylinders with diameter of 7.8 ± 0.2 mm and height of 1.9 ± 0.1 mm. The strut size and spacing of the scaffolds were 230 ± 11 μm and 140 ± 7 μm, respectively. Individual struts had an estimated modulus of 91.93 ± 23.32 MPa. In vitro cell studies demonstrated the ability of 3D printed ICIE16 bioactive glass scaffolds to stimulate hBMSC differentiation down an osteogenic pathway and that cell attachment to the surface is important, in addition to ion release. Osteogenic gene expression was enhanced when hBMSCs were cultured directly on the scaffolds compared to culture on tissue culture plastic in the presence of the dissolution products of the scaffolds. Osteogenic differentiation occurred in the presence of the scaffolds, without osteogenic supplements, but culturing in osteogenic supplements did offer synergistic acceleration of bone production in vitro.

## Data Availability

Raw data is available on request from rdm-enquiries@imperial.ac.uk.

## Abbreviations

hBMSCs: human bone marrow derived stromal cells
HOBs: human osteoblasts
IGF: insulin-like growth factor
ATP: adenosine triphosphate
ALP: alkaline phosphatase
FBS: foetal bovine serum
PBS: phosphate-buffered saline
α-MEM: minimum essential medium alpha modification
XRD: X-ray diffraction
HCA: hydroxyapatite
SEM-EDS: scanning electron microscopy with energy dispersive spectroscopy
